# Construction of Whole Genome Radiation Hybrid Panels and Map of Chromosome 5A of Wheat Using Asymmetric Somatic Hybridization

**DOI:** 10.1371/journal.pone.0040214

**Published:** 2012-07-16

**Authors:** Chuanen Zhou, Wei Dong, Lu Han, Jiajie Wei, Li Jia, Yang Tan, Daying Zhi, Zeng-Yu Wang, Guangmin Xia

**Affiliations:** 1 Key Laboratory of Plant Cell Engineering and Germplasm Innovation, Ministry of Education, School of Life Science, Shandong University, Jinan, Shandong, P.R. China; 2 Forage Improvement Division, Samuel Roberts Noble Foundation, Ardmore, Oklahoma, United States of America; University of Guelph, Canada

## Abstract

To explore the feasibility of constructing a whole genome radiation hybrid (WGRH) map in plant species with large genomes, asymmetric somatic hybridization between wheat (*Triticum aestivum* L.) and *Bupleurum scorzonerifolium* Willd. was performed. The protoplasts of wheat were irradiated with ultraviolet light (UV) and gamma-ray and rescued by protoplast fusion using *B. scorzonerifolium* as the recipient. Assessment of SSR markers showed that the radiation hybrids have the average marker retention frequency of 15.5%. Two RH panels (RHPWI and RHPWII) that contained 92 and 184 radiation hybrids, respectively, were developed and used for mapping of 68 SSR markers in chromosome 5A of wheat. A total of 1557 and 2034 breaks were detected in each panel. The RH map of chromosome 5A based on RHPWII was constructed. The distance of the comprehensive map was 2103 cR and the approximate resolution was estimated to be ∼501.6 kb/break. The RH panels evaluated in this study enabled us to order the ESTs in a single deletion bin or in the multiple bins cross the chromosome. These results demonstrated that RH mapping via protoplast fusion is feasible at the whole genome level for mapping purposes in wheat and the potential value of this mapping approach for the plant species with large genomes.

## Introduction

Wheat (*Triticum aestivum* L.; 2n = 6× = 42; A, B and D genome), which contains 16,000 Mb of DNA organized into three genomes, is the most widely grown crop in the world. However, the progress of systematic localization of genes with agriculturally important traits had been hampered due to its large genome size and high frequency of repetitive DNA. To identify closely associated molecular markers for marker-assisted selection and positional cloning, a number of genetic maps were developed based on meiotic recombination. However, the recombination along the chromosome arms in many plant species is not evenly distributed [1], [2], and thus limits opportunities to identify functionally relevant genes. In wheat, it was estimated that about 30% of the total number of genes are in recombination-poor regions and thus the reduced recombination rates resulted in limited resolution of the genetic map [3]. Additionally, the genetic distances may not reflect the physical distance. For example, 1 cM equals 118 kb in regions of high recombination and 22,000 kb in regions of low recombination in wheat [4]. Therefore, it is difficult to investigate the whole genome of wheat, especially in regions with low recombination, due to the limited ability to develop high-resolution genetic maps [5]. Recently, many physical mapping approaches adopting some new mapping strategies were developed to facilitate the localization of genes and molecular markers by providing the actual physical location on the chromosome [5], [6], [7], [8]. The sequencing and assembly of large genomes of plant species such as wheat also requires physical maps with high resolution [6]. In addition, a collection of deletion lines was generated and used for placing the genes and ESTs [1]. Using about 440 deletion stocks, over 16,000 ESTs were placed onto wheat chromosomes by deletion bin mapping [9]. Although this is an effective way to place markers on specific chromosomal regions, the order among the ESTs located in a single bin is yet to be determined because these bins cover the large deletions [9], [10].

Radiation hybrid (RH) mapping is a recombination-independent mapping approach, relying on radiation-induced chromosome breakage [11]. This method has been used successfully in mammalian species for high resolution mapping via asymmetric somatic hybridization [12], [13]. In the RH mapping system, irradiated donor chromosomes are integrated randomly into a recipient genome by cell fusion to produce the hybrid cell lines. A panel of this kind of cell lines are then used for mapping [12]. The resolution of the RH map can be adjusted to some extent by varying the radiation dosages, which is a unique advantage of this method [14], [15]. In the early RH system, only one chromosome could be mapped at a time [12]. Then, whole-genome radiation hybrid (WGRH) mapping was developed to generate whole-genome radiation hybrids and allow all chromosome to be mapped using a single RH panel [16]. Consequently, the success of RH mapping was applied in other animal species [17], [18], [19], [20].

The first RH map in plants was developed for mapping maize chromosome 9 using an oat-maize addition line [21]. The barley RH panel was constructed to chromosomally allocate EST clones using protoplast fusion for *in vitro* rescue of donor chromosome fragments [22], [23]. The WGRH mapping, a similar method established in animals, was used in cotton [24], [25]. This method was named wide-cross radiation hybrid (WWRH) mapping and allowed the placement of 102 SSR markers onto chromosomes [25]. To date, the highest resolution of an RH map in plants is of the wheat chromosome 1D (D genome) [10]. The RH panel was developed in a durum wheat (*Triticum turgidum* L. AABB) line carrying chromosome 1D of *T. aestivum* L. (AABBDD), containing the *species cytoplasm-specific* (*scs^ae^*) gene [26]. The first attempt to implement RH mapping in this system allowed the localization of *scs^ae^* gene and 39 molecular markers on the chromosome 1D. Then, the same panel was analyzed with 378 markers, revealing that the resolution of this RH panel was about 199 kb/break [10].

Asymmetric somatic hybridization by protoplast fusion was proven to be a successful method to transfer donor genetic materials into a recipient species and create hybrids across sexual borders [27], [28]. Different techniques for the induction of asymmetric hybrids have been developed [29], [30], [31], [32]. In most asymmetric fusions, the donor protoplasts are treated with ionizing irradiation (X-ray or gamma-ray) by varying the radiation dose to produce asymmetric hybrids [33], [34]. Compared with ionizing irradiation, ultraviolet light (UV) radiation treatment can also induce the elimination of the donor genetic materials and affect the frequency of surviving hybrids in a dose-dependent manner [30], [31]. Because of its easy access and convenience, many intergeneric or intertribal asymmetric somatic hybrids were produced by UV radiation [27], [28], [35].

*Bupleurum scorzonerifolium* Willd. (2n = 12; Umbelliferae, dicotyledon) is a traditional Chinese herb used to treat influenza, fever, malaria, and menstrual disorders and its regeneration system was constructed in our lab [36]. Due to its remote phylogenetic relationship to wheat and unique stability of the genome in long-term subculture [37], [38], [39], we used *B. scorzonerifolium* as the recipient species to construct the whole genome RH panel of wheat. In this study, seven types of asymmetric somatic hybrids between wheat and *B. scorzonerifolium* were induced by the irradiation of UV and gamma-ray. We investigated the possibility of using wheat / *B. scorzonerifolium* asymmetric somatic hybrids to construct an RH map by evaluating the retention frequencies of SSR markers of wheat in the hybrids. Based on these analyses, two wheat WGRH panels with different numbers of hybrids were obtained. Taking wheat chromosome 5A as an example, we constructed a comprehensive RH map using SSR markers, ordered 25 previously deletion-binned but unordered ESTs and positioned 46 ESTs along the chromosome 5AL via RH mapping approach. These results demonstrated that RH mapping via cell fusion is suitable for mapping at the whole genome level in the plant species with large genomes.

## Materials and Methods

### Protoplasts Origin and Isolation

Embryogenic calli of common wheat (*T. aestivum* L. cv. Jinan 177) were induced from excised immature embryos on MB medium [40]. Yellow, granular calli were subcultured for two years. The inducement and culture of *B. scorzonerifolium* calli were performed as previously described [36]. For the fusion experiments, the protoplasts of wheat and *B. scorzonerifolium* were isolated respectively from their calli as previously described [36], [38].

### Protoplast Irradiation, Fusion and Culture

Two types of radiations UV and gamma-ray were used in the experiments. For UV irradiation, wheat protoplasts were placed inside 3.5 cm diameter Petri dishes in a single layer, irradiated with UV at an intensity of 300 µW/cm^2^ for 30 s, 60 s, 120 s and 180 s. For gamma-ray irradiation, wheat cells were irradiated with the dose of 3 Krad, 5 Krad and 7 Krad at a dose rate of 1.8 Krad/h using a ^60^Co gamma source before protoplast isolation. Irradiated wheat protoplasts as donors were fused with *B. scorzonerifolium* protoplasts as receptor at a ratio of 1∶1 using polyethylene glycol (PEG) [40]./Seven asymmetric hybridization combinations were performed and the symmetric hybridization between wheat and *B. scorzonerifolium* was carried out as control combination (Table 1). In addition, cultures of both untreated parental protoplasts were also used as controls. The process of protoplast fusion and culture were performed as described previously [38].

**Table 1:** The somatic hybridization combinations between wheat and *B. scorzonerifolium*.

Ray	Combination symbol	Donor (radiation treatment) × Recipient
None	WB	Wheat × *B. scorzonerifolium*
UV	WB30	Wheat (UV for 30 s) × *B. scorzonerifolium*
	WB60	Wheat (UV for 60 s) × *B. scorzonerifolium*
	WB120	Wheat (UV for 120 s) × *B. scorzonerifolium*
	WB180	Wheat (UV for 180 s) × *B. scorzonerifolium*
Gamma-ray	WB3K	Wheat (gamma-ray for 3 Krad) × *B. scorzonerifolium*
	WB5K	Wheat (gamma-ray for 5 Krad) × *B. scorzonerifolium*
	WB7K	Wheat (gamma-ray for 7 Krad) × *B. scorzonerifolium*

### Hybrid Nature Identification

For the identification of the hybrid nature of the fusion products, seven random RAPD primers (Sangon, China) were screened, three of which primers, namely *OPV-07* (5′- GAAGCCAGCC), *OPJ-19* (5′- GGACACCACT), *OPA-19* (5′- CAAACGTCGG), could distinguish the fusion partners. The amplified DNA fragments were separated by electrophoresis in 1.5% agarose gels, stained with ethidium bromide, and photographed under UV using Syngene gel imaging system (Syngen, USA). A λDNA/*Eco*RI+*Hin*dIII marker (Sangon, China) was used as a molecular weight standard. For chromosomes analysis, fresh calli of the parents and putative hybrid clones were pre-treated and pressed for chromosome spreading on glass slide. Genomic *in situ* hybridization (GISH) was performed following the methods described previously [41]. Total genome DNA of wheat was labelled as a probe using digoxigenin and detected with FITC (yellow-green). The chromosomes were counterstained with propidium iodide (PI).

### Microsatellite Marker Analysis

All the hybrid clones obtained from eight combinations were tested for the retention of donor chromatin using 42 microsatellite loci mapping to the short and long arms of each chromosome of wheat. The amplified SSR products were resolved on 6% PAGE, and banding patterns were visualized using silver staining protocol as described previously [42]. The 100 bp DNA ladder (Sangon, China) was used as a molecular weight standard.

### Map Construction

The CarthaGene program was used to build the RH map for wheat chromosome 5A, 5AL and order the ESTs in bin C-5AL10-0.57*. The program is available at http://www.inra.fr/internet/Departements/MIA/T/CarthaGene. The two-point LOD threshold of four and a distance of 100cR were set for the significant linkage groups. The number of chromosome breaks was calculated using the *mapocb* commond. Since all SSR and EST markers are known from wheat chromosome 5A, the comprehensive map included all markers was generated as described previously [10].

## Results

### Hybrid Verification, Culture and Differentiation

**Figure 1:**
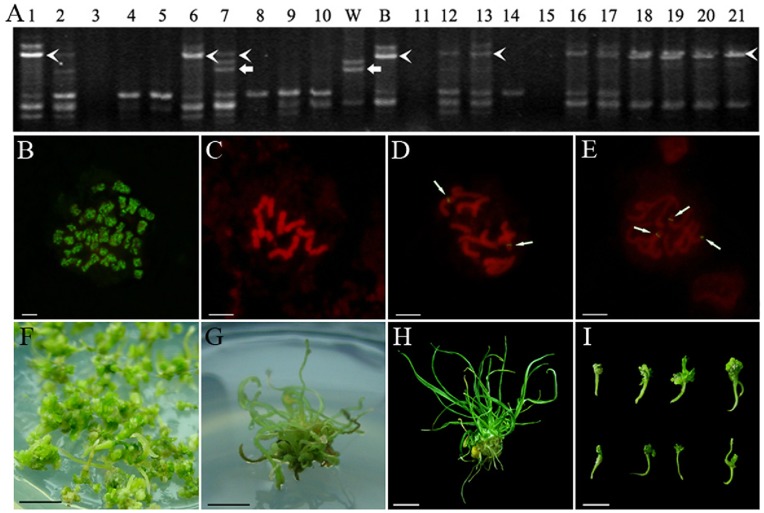
Identification of hybrid cell clones. A: RAPD amplification pattern generated using primer *OPV-07*. W: Wheat; B: *B. scorzonerifolium*; 1–21: Regenerated cell clones; arrowhead: *B. scorzonerifolium* characteristic band; arrow: Wheat characteristic band; cell clone No.7 was identified as a hybrid due to the presence of both parental specific bands. B-E: GISH analysis. B: Wheat; C: *B. scorzonerifolium*; D: Hybrid cell clone No.10 in WB5K showing two chromosomal fragments of wheat inserted into the genome of *B. scorzonerifolium*; E: Hybrid cell clone No.7 in WB60 showing three recombined chromosomes; arrow: Chromosomal fragments of wheat; *Bar*: 5 µm. F–I: Differentiation of hybrid cell clones. F: The shoots regenerated from hybrid clone No.64 in WB120; G: The plantlet of intermediate type regenerated from hybrid cell clone No.88 in WB60; H: The plantlet resembled *B. scorzonerifolium* regenerated from hybrid cell clone No.41 in WB60; I: Diverse morphology of regenerated shoots in combination WB120; *Bar*: 1cm.

A total of 665 single cell clones were obtained from seven asymmetric combinations and one symmetric control combination after 45–50 days of culture (Table 1). These clones were transferred to the proliferation and differentiation medium. All the single cell clones were similar to *B. scorzonerifolium* callus. Some of them regenerated plantlets with phenotypes of *B. scorzonerifolium* or intermediate between both parents (Figure 1F-I). As the control, protoplasts of wheat could only grow to cell clusters but not calli. Protoplasts of *B. scorzonerifolium* showed the capability to differentiate into green spots but could not regenerate plantlets. All single cell clones were analyzed using RAPD markers. The clones possessing both wheat and *B. scorzonerifolium* specific band(s) were identified as hybrids (Figure 1A). Among the 665 cell clones, 163 were recognized as hybrids on the basis of RAPD profiles (Table 2). The hybrid nature of regenerated cell clones was also confirmed by genome *in situ* hybridization (GISH) analysis. From the GISH karyotypes, the chromosome numbers in 90% of the hybrid analyzed clones showed 2n = 12, with 1–3 wheat chromosomal fragments (Figure 1B-E).

**Table 2:** Hybrid number,frequency and differentiation capacity of different hybridization combinations.

Ray type	Combination symbol	Number of regenerated cell clones	Number of hybrid clones	Hybrid frequency		Differentiation		
				%	Average	+^a^ Number of hybrids (%)	++^b^ Number of hybrids (%)	+++^c^ Number of hybrids (%)
None	WB	57	8	14.0	–	6(75.0)	2(25.0)	0(0)
UV	WB30	82	14	17.1	21.4	11(78.6)	3(21.4)	0(0)
	WB60	140	41	29.3		32(78.0)	7(17.2)	2(4.8)
	WB120	142	36	25.4		29(80.6)	7(19.4)	0(0)
	WB180	102	14	13.7		11(78.6)	3(21.4)	0(0)
Gamma-ray	WB3K	55	15	27.3	35.5	12(80.0)	3(20.0)	0(0)
	WB5K	58	24	41.4		20(83.3)	4(16.7)	0(0)
	WB7K	29	11	37.9		9(81.8)	2(18.2)	0(0)

a no differentiation ability, ^b^differentiate into leaves and shoots, ^c^differentiate into complete plantlet.

Seven asymmetric combinations were divided into two groups: UV and gamma-ray combination groups, according to the different radiation treatments (Table 1). In total 155 hybrids were obtained and the hybrid frequencies generated from asymmetric combinations ranged from 13.7% to 41.4%. The trend of average hybrid frequency changed from low to high and then back to low when the radiation dosages of UV and gamma-ray increased (Table 2). Compared with the wide range of hybrid frequencies, the differentiation frequencies of each asymmetric hybridization combination ranged from 16.7% to 21.4 % and were independent of the UV or gamma-ray dosage. As for the control combination WB, it had low hybrid frequency (14.0%) but high differentiation frequency (25.0%) (Figure 1F-I; Table 2).

### Retention Frequencies of Wheat Genome in Hybrid Clones

**Figure 2:**
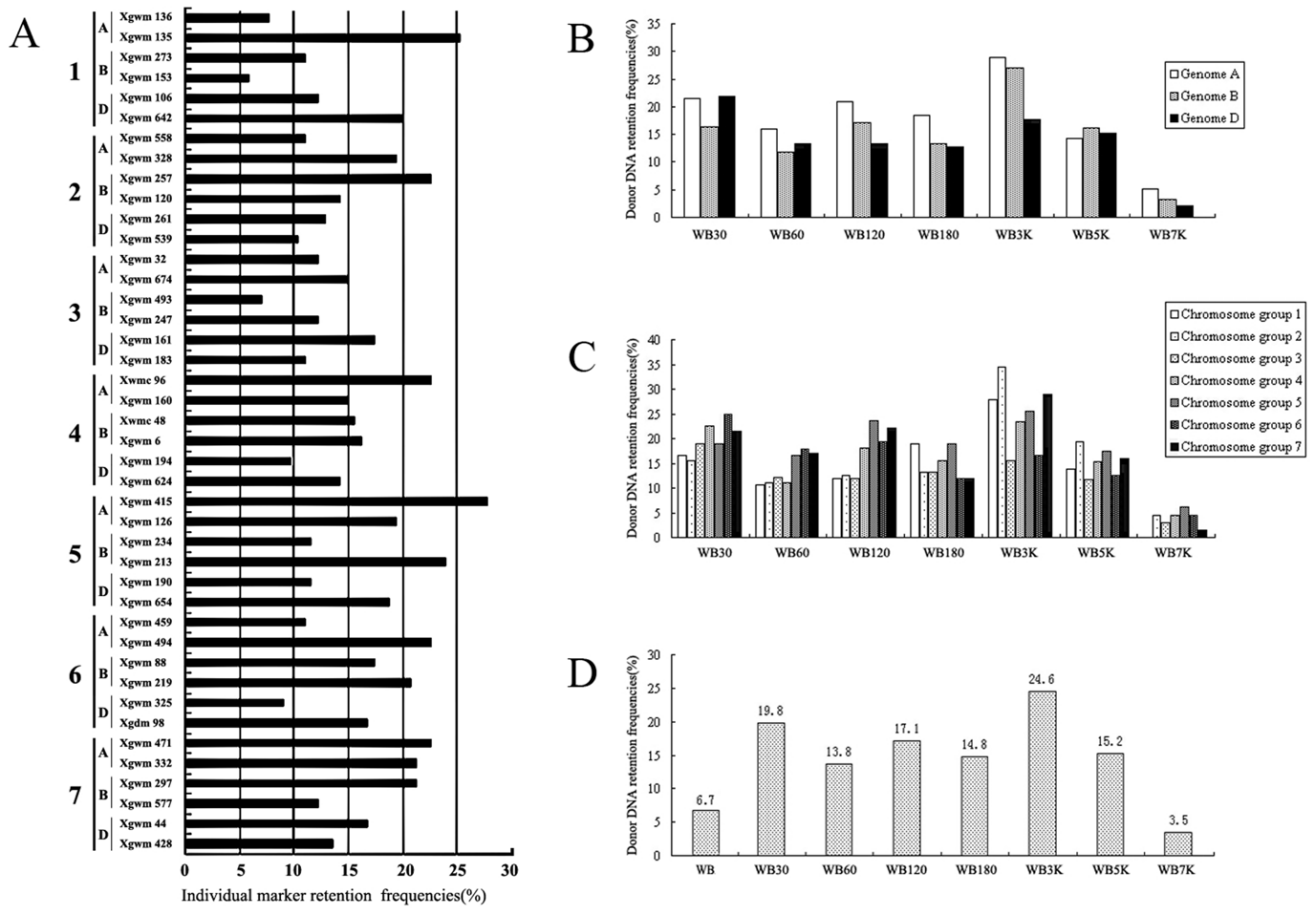
Individual marker retention frequency and donor DNA retention frequency of wheat in radiation hybrids. A: Individual marker retention frequencies in 155 radiation hybrids derived from seven asymmetric hybridization combinations. Forty-two microsatellite loci across the wheat genome are listed to the left of the figure. B–D: Donor DNA retention frequency of wheat in hybrids. B: Average donor DNA retention frequencies of A, B, D genome of wheat in each hybridization combination; C: Average donor DNA retention frequencies of seven chromosome groups of wheat in each of hybridization combinations; D: Average donor DNA retention frequencies of seven asymmetric hybridization combinations and control combination.

All 155 hybrid clones were analyzed using 42 SSR markers, providing genome-wide coverage of the wheat genome to estimate the distribution of wheat chromatin in *B. scorzonerifolium* background. The wheat specific SSR markers were not able to produce PCR amplicons in the *B. scorzonerifolium* template. Individual marker retention frequencies in 155 hybrid clones was calculated and varied from 5.8 to 27.7% with an average retention frequency of 15.5 % (n = 42) (Figure 2A). Of the 42 SSR markers, two (*Xgwm135* and *Xgwm415*) were retained more frequently than the others. 42 markers were present in all asymmetric hybrids, but different markers were absent in different individual hybrids. A total of 88.1% of the 42 markers had retention frequencies between 10% and 30%. Donor DNA retention frequency (DF) represents the number of markers retained within an individual hybrid. In seven asymmetric hybridization combinations, the average DFs for three progenitor wheat genomes (A, B and D) and seven chromosome groups of wheat were calculated respectively. Friedman two-way ANOVA test analysis indicated that no significant statistical difference existed among the three subgenomes and seven chromosome groups of wheat in the same hybridization combinations although there was some visible difference of DFs (Figure 2B, C). However, as for the gamma-ray combination group, there was a decrease in the average DFs with the radiation dose enhanced, showing a dose-dependent effect (Figure 2D). The same effect was observed in the UV combination group with the exception of WB60. It was noticed that the decrease in DFs in the UV combination groups was less than that of the gamma-ray combination groups (Figure 2D).

### The Radiation Induced Breakages and RH Map of Chromosome 5A

A random set of 92 of the 155 hybrid clones were selected to represent the Radiation Hybrid Panel of Wheat I (RHPWI). To compare the efficiency using different numbers of hybrid clones for the map construction, we produced another 29 hybrid clones between wheat and *B. scorzonerifolium* in addition to the 155 hybrid clones to represent the Radiation Hybrid Panel of Wheat II (RHPWII). Theoretically, both RHPWI and RHPWII could be used for the map construction of any chromosomes of wheat because that these two panels were produced with the segmentation of whole wheat genome by irradiation. Chromosome 5A of wheat was found to have large effects on both grain size and shape-related parameters [43], [44]. Therefore, we selected 68 SSR markers located in 5A chromosome of wheat for genotyping to explore the mapping feasibility of the panels .

**Figure 3:**
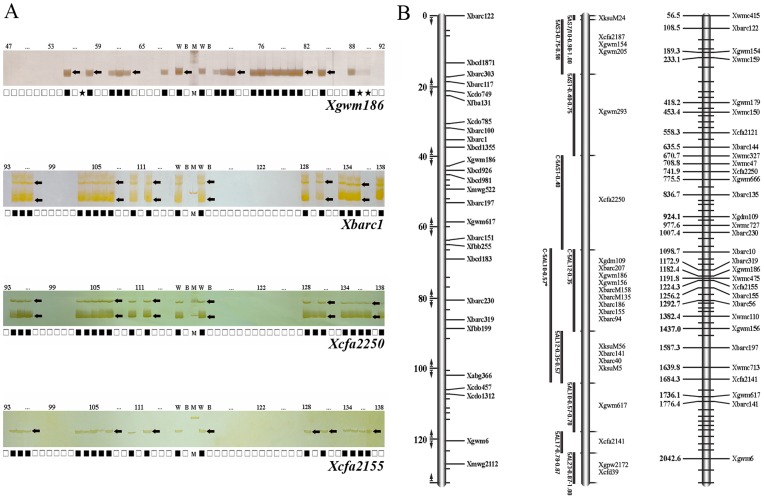
Genotyping of nuclear SSR markers and the RH map of wheat chromosome 5A. A: Genotyping of SSR loci *Xgwm186*, *Xbarc1*, *Xcfa2250* and *Xcfa2155* in RH panel. W: Wheat; B: *B. scorzonerifolium*; 47–138: Radiation hybrids; *M*: 100 bp DNA ladder; arrow: SSR-PCR products of wheat; Solid squares represent retained markers, open squares represent eliminated markers and asterisks represent undetermined data. B: Wheat chromosome 5A RH map (right) as compared with the genetic (left) and the deletion map (middle). The black lines on the deletion map refer to bins assigned to the chromosome 5A. The RH map has an estimated total length of 2103 cR covered by 68 SSR markers.

For RHPWI, a total of 6256 SSR loci (68×92) were detected, among which 2102 SSR loci were retained in the panel and 4064 SSR loci were not (Figure 3A). The rest of SSR loci could not be identified due to the presence of undetermined bands. The average marker retention frequency in RHPWI was 33.59% and all hybrid clones had at least one marker. In the same way, we indentified 12512 SSR loci (68×184) in RHPWII using the same SSR markers, among which 4252 SSR loci were retained and 8144 SSR loci were not. The average marker retention frequency in RHPWII was 33.98%. Out of 184 hybrid clones, 10 had lost all 68 SSR loci.

The resolution of the RH map can be directly assayed by estimating the average distance between breaks [10]. Based on the SSR genotyping results, the numbers of obligate breaks along chromosome 5A calculated by CarthaGene was 1557 and 2034, in RHPWI and RHPWII respectively. Additionally, the approximate DNA content of chromosome 5A is about 781 Mb according to the relative length of somatic metaphase chromosomes and nuclear genome size of wheat genome [45], [46]. Assuming the SSR markers are randomly distributed along the chromosome 5A, the resolution of the panels could be calculated roughly by estimating the average distance between radiation-induced breaks. Therefore, the resolutions of RHPWI and RHPWII were approximate 501.6 kb (781 Mb/1557 obligate breaks) and 383.9 kb (781 Mb/2034 obligate breaks), respectively.

To determine the relationship between cR and the physical distance on chromosome 5A, the comprehensive RH map was constructed using a lower confidence (LOD 2.0) since all SSR markers belong to chromosome 5A. This strategy has been used for RH map construction of chromosome 1D in wheat [10]. The data used for mapping are derived from RHPWII because of its higher resolution. The results showed that the distance of RH map of chromosome 5A was 2103cR (Figure 3B, see Table S1). Since the size of chromosome 5A is estimated at about 781 Mb, 1cR equals to 371 kb in this RH map.

### Alignment of RH Map to Physical Map

**Figure 4:**
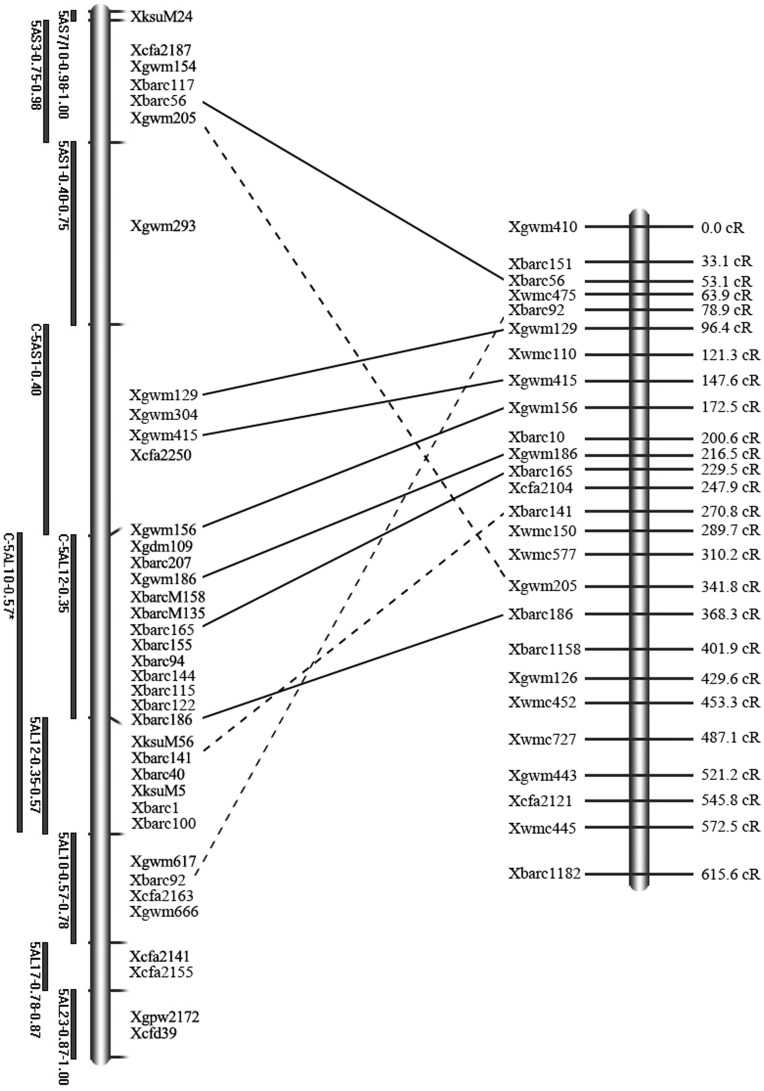
Alignment of RH map to physical map. Comparison between wheat chromosome 5A RH linkage group (right) with deletion map (left). The solid lines between markers show the consistent order between RH map and deletion map. The broken lines show the inconsistent marker order between two maps.

The linkage groups were generated at a two-point LOD score of 4.0 by group command in CarthaGene. A total of 14 linkage groups and unlinked markers were identified. To further estimate the reliability of the RH mapping population, the biggest linkage group was chosen and further generated a framework map which contained 26 SSR markers and covered a total of 615.6cR. Then, this map was compared with the known physical map of chromosome 5A [47]. Out of 26 markers, 10 were aligned with the deletion map (Figure 4). The order of most of markers (7/10) is consistent with that of makers in physical map, indicating that the RH panels constructed by protoplast fusion have the ability for mapping.

### Ordering the 25 ESTs within the Bin C-5AL10-0.57* on Chromosome 5A

Bin C-5AL10-0.57* contained the centromeric region and the long arm of chromosome 5A based on the deletion map. In this bin, 25 ESTs with unknown order between each other were selected for evaluating the ordering ability of markers within this single linkage group. This region was compared with rice genome by searching 25 ESTs against rice genome using program BlastX at RGAP (Rice Genome Annotation Project: http://rice.plantbiology.msu.edu/analyses_search_blast.shtml) (see Table S2). Out of 25 ESTs, 8 are located in rice chromosomes 9 and 12 (highlighted with yellow color). Some ESTs with high hit score are located in rice chromosome 2, 3, 5 and 7 (highlighted with green color). These data probably indicated that the different structure of pericentromiric region (at least in bin C-5AL10-0.57*) between wheat and rice.

**Figure 5:**
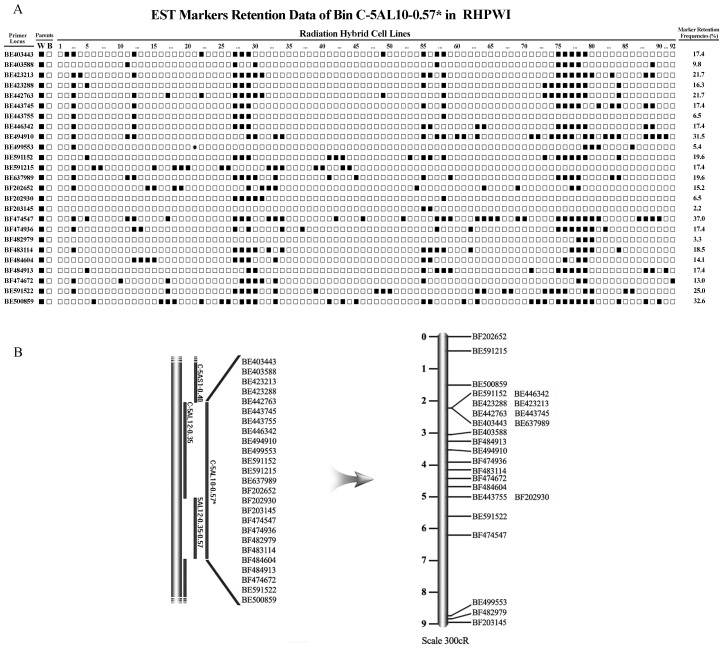
Genotyping and RH mapping of 25 EST loci. A: Retention frequencies of 25 EST loci of bin C-5AL10-0.57* in the Radiation Hybrid Panel of Wheat I (RHPWI). Twenty-five EST loci are listed to the left of the figure. Solid squares represent retained markers, open squares represent eliminated markers and asterisks represent undetermined data. Individual marker retention frequencies of the panel are listed to the right of the figure. W: Wheat; B: *B. scorzonerifolium*; 1–92: Radiation hybrids. B: The order of 25 ESTs in the bin C-5AL10-0.57^*^ using the RH mapping approach on RHPWI (right). The black lines on the deletion map (left) refer to bins assigned to chromosome 5A.

The PCR products amplified from each of the 25 ESTs was a single fragment. Based on the genotyping results from RHPWI, 11 of the 92 hybrid clones were missing all 25 ESTs, while 9 hybrid clones retained almost all the ESTs. All 25 ESTs were lost at least once, indicating that these ESTs markers were retained in RHPWI randomly (Figure 5A). The average marker retention frequency in RHPWI was 17.0%. A total 460 obligate breaks were estimated in this region by CarthaGene (see Figure S1). The RH map of bin C-5AL10-0.57* was constructed and 25 ESTs were divided into two linked groups and 15 individual ESTs. The first linked group consisted of BE591152, BE423288, BE442763, BE403443, BE446342, BE423213, BE443745, BE637989 and the second one consisted of BE443755 and BF202930 (Figure 5B). The results showed that radiation treatment could induce the breakages anywhere along the chromosome. The RH mapping approach has the ability to order the markers within a single bin even in centromeric regions.

### Alignment of RH Map to a Virtual Gene Order of Chromosome 5AL

**Figure 6:**
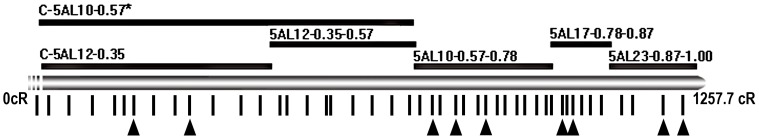
Consistency between RH map and virtual gene order of chromosome 5AL. The black lines above refer to deletion bins assigned to the chromosome 5AL. Each EST is represented by a bar and positioned according to the results of RH mapping. In total 46 ESTs are ordered on chromosome 5AL. The ESTs marked by arrowheads showed the inconsistent order with the virtual gene order.

A recent report provided the sequencing data of wheat chromosome 5A [48]. In this study, a genome zipper of wheat chromosome 5A was generated. It was integrated with the physical map, in which 47 ESTs mapped on the 5AS zipper and 139 ESTs mapped on the 5AL zipper. To further demonstrate the mapping capability of the RH panel, 63 ESTs were chosen from 5AL zipper for RH mapping. All these selected ESTs were from non-overlapping bins and showed the consistency between virtual gene order and the deletion bin map [48]. The linkage groups were generated at a two-point LOD score of 4.0 by CarthaGene. A comprehensive RH map contained 46 ESTs was constructed following the process mentioned above since all ESTs are known from chromosome 5AL (see Table S3). The results showed that the average marker retention frequency of 46 ESTs in RHPWI was 25.1% and a total 1134 obligate breaks were estimated in 5AL. This RH map of chromosome 5AL covered a total of 1213.6cR. To evaluate the quality of RH map, the consistency of gene order between RH map and 5AL zipper was verified (Figure 6). Out of 46 ESTs, the order of 37 ESTs (80%) is consistent with the virtual gene order, indicating the accuracy of the RH mapping in chromosome 5AL.

## Discussion

### Random Introgression of Wheat Chromatin Induced by UV and Gamma-ray

In these experiments, the protoplasts of *B. scorzonerifolium* could only form green spots and the protoplasts of wheat could only develop into cell clusters, so the regenerated hybrid shoots and plantlets (Figure 1F-I) are the results of genetic complementation of parents as previously reported [27], [41]. Based on the analysis of DFs in different asymmetric somatic combinations, the amount of wheat chromatin transferred to the *B. scorzonerifolium* genome was obviously different, but the irradiation types and dosages did not affect the differentiation ability of radiation hybrids (Table 2). No significant statistical differences of DFs among the three subgenomes or seven chromosome groups of wheat were observed in the radiation hybrids (Figure 2). These data suggest that the chromatin of wheat was introgressed randomly into the *B. scorzonerifolium* genome. Random introgression of donor genetic materials induced by irradiation was also reported previously [23] and genetic constitution in each of hybrid clones is rather different even if they were derived from same hybridization combination [23], [38], [49]. Therefore, random introgression of donor genetic materials represents an advantage to construct a whole genome radiation hybrid panel in plants.

### Construction of RH Panel by Asymmetric Somatic Hybridization

The reports of RH mapping in plants are limited [10], [21], [22], [23], [24], [25], [50], due in part to the difficulty of finding appropriate recipient cell lines to rescue irradiation-fragmented donor chromosomes [24]. In animals, the donor chromosome fragments can be rescued by somatic hybridization [13], [19]. In plants, irradiated chromosomal segments can also be recovered *in vitro* [22], [23], [38]. Alternatively, the RH panel can be constructed by using the seeds or pollen as the target of irradiation, then self-pollination or sexually crossing with a recipient plant for the retention of donor chromosome fragments [10], [21], [24], [25], [50]. The construction of RH panels by *in vivo* rescue is dependent on the special addition line or closely related species. For example, the maize chromosome 9 RH panel was constructed using an oat-maize addition line. But the similar systems were difficult to develop in other species [21]. The “wide-cross whole-genome radiation hybrid” panel from cotton was developed by the cross between the donor species *G. hirsutum* and the recipient species *G. barbadense* which are in the same genus [24], [25]. However, a close relationship between the parents will decrease the level of marker polymorphism, and isolation of the donor chromosome fragments becomes difficult [51]. The first *in vitro* whole genome radiation hybrid panel was developed by introgression of barley (*Hordeum vulgare*) genome fragments into tobacco protoplasts [23]. The advantage of somatic hybridization in plant is to by-pass barriers to sexual hybridization to produce hybrids [27]. In our previous studies, we identified *B. scorzonerifolium* as a suitable host for studying the wheat genome due to its unique genetic stability and the distant phylogenetic relationship with wheat (inter-family) [38]. Therefore, in this RH mapping system, the distant phylogenetic relationship between the parents allows us to order the genes and molecular markers based on the presence or absence of markers in a panel of hybrid cells without the need of polymorphism of them. As a result, the markers with low polymorphism between the parental lines in wheat genetic map could be placed in the RH mapping system.

### The Potential Ability of Increasing Retention Frequency, Patterns and Resolution in an *in Vitro* RH Panel

In RH mapping of humans and animals, 10 to 50% of donor genome retention is considered suitable for mapping [14], [52]. In plants, the average marker retention frequencies of RH panels obtained by *in vivo* rescue are higher than 70% [10], [21], [25]. In addition, high donor retention frequency and limited level of chromosome breakage was also found in the maize chromosome 9 RH panel. The reason is that the developmental capability of irradiated seeds is required for this mapping system [21]. However, the RH panels developed *in vitro* showed a relatively lower donor retention frequencies. For example, 24.67% in the barley RH panel [23] and the wheat RH panel (RHPWI, 33.59%; RHPWII, 33.98%; this study), which are similar to the RH panels in animals, suggesting that the *in vitro* method is suitable for plant RH mapping. On the other hand, marker retention pattern may be more important than chromosomal retention frequency for high resolution RH mapping [24], [25]. The variation of marker retention patterns requires high levels of chromosomal breakage which is partially dependent on the radiation dosages. A continuous increase in the radiation dosage resulted in a significant increase in the retention patterns instead of marker retention frequency [21], [24]. However, high radiation dosages are known to reduce the survival frequency of irradiated seeds or pollen in the RH mapping system using *in vivo* rescue [21], [25]. However, high radiation dosages can be used in somatic hybridization because the RH panel was developed *in vitro*, implying that the numbers of retention patterns could be potentially increased in this RH mapping system.

The resolution of RH panel is dependent on the incremental induction of chromosome breakage [52]. However, if the amount of chromosome breakage is limited, enriching the panel with radiation hybrids might provide an alternative way to obtain higher resolution [21], [53]. The mapping resolution will also increase if more markers are assayed on the panel for the construction of an RH map due to the ability to detect a higher number of breaks. In this experiment, although the resolutions are roughly calculated by dividing the number of obligate breaks with the approximate size of chromosome 5A, they are comparable each other because they are estimated by the same markers and in the same RH mapping system. The mapping resolution was about 501.6 kb/break in RHPWI (92 hybrids) and 383.9 kb/break in RHPWII (184 hybrids), indicating that increasing the hybrid numbers translates to a greater map resolution. In our study, a RH map contained 46 ESTs was constructed using RHPWI. This map showed 80% consistency of gene order with the virtual gene order by alignment to a 5AL genome zipper. It is possible that their collinearity could be improved if RHPWII instead of RHPWI was used for generating RH map of 5AL since more chromosome breaks will be detected if a higher number of hybrids are analyzed.

### Combined RH Mapping and Deletion Mapping for Locating the EST

To date, over 16,000 ESTs were mapped in the wheat genome using the deletion lines representing a total of 159 chromosomal bins [1], [9]. Within these ESTs, about 2500 were located in the wheat homoeologous group 5 and 34% (850 ESTs) were located on chromosome 5A. There are 9 bins in the deletion lines used for mapping wheat homoeologous group 5, resulting in an average of 94 unordered ESTs within a single bin. The bin numbers decrease from telomere to centromere in the chromosomes of group 5 [54], making it difficult to map the markers in the regions around the centromere of wheat homoeologous group 5 by deletion lines. In this study, bin C-5AL10-0.57* was used for mapping to explore the ability of ordering the ESTs in centromeric regions using RHPWI because it comprised the portions of centromere. Based on the genotyping of 25 ESTs, the average marker retention frequency is estimated at 17%, which is lower than that of SSR markers in the same panel (33.59%). 460 obligate breaks were detected in this region, suggesting that our RH panel could be used for mapping markers in small region.

## Supporting Information

Figure S1 Two-points breakage ratio matrix of the 25 EST loci of bin C-5AL10-0.57*.
(DOCX)

Table S1 The RH map of 68 microsatellite loci of wheat chromosome 5A.
(DOCX)

Table S2 The putative rice homologs of 25 ESTs of bin C-5AL10-0.57*.
(DOCX)

Table S3 The RH map of 46 ESTs of wheat chromosome 5AL.
(DOCX)

## References

[1] Endo TR, Gill BS (1996). The deletion stocks of common wheat.. Journal of Heredity.

[2] Sandhu D, Gill KS (2002). Gene-containing regions of wheat and the other grass genomes.. Plant Physiol.

[3] Erayman M, Sandhu D, Sidhu D, Dilbirligi M, Baenziger PS (2004). Demarcating the gene-rich regions of the wheat genome.. Nucleic Acids Resarch.

[4] Masoudi-Nejad A, Nasuda S, Bihoreau MT, Waugh R, Endo TR (2005). An alternative to radiation hybrid mapping for large-scale genome analysis in barley.. Mol Gen Genet.

[5] Kalavacharla V, Hossain K, Riera-Lizarazu O, Gu Y, Vales MI (2009). Radiation hybrid mapping in crop plants.. Advances in Agronomy.

[6] Fleury D, Luo MC, Dvorak J, Ramsay L, Gill BS (2010). Physical mapping of a large plant genome using global high-information-content-fingerprinting: the distal region of the wheat ancestor Aegilops tauschii chromosome 3DS.. BMC Genomics.

[7] Paux E, Sourdille P, Salse J, Saintenac C, Choulet F (2008). A physical map of the 1-gigabase bread wheat chromosome 3B.. Science.

[8] Luo MC, Ma Y, You FM, Anderson OD, Kopecky D (2010). Feasibility of physical map construction from fingerprinted bacterial artificial chromosome libraries of polyploid plant species.. BMC Genomics.

[9] Qi LL, Echalier B, Chao S, Lazo GR, Butler GE (2004). A chromosome bin map of 16,000 expressed sequence tag loci and distribution of genes among the three genomes of polyploid wheat.. Genetics.

[10] Kalavacharla V, Hossain K, Gu Y, Riera-Lizarazu O, Vales MI (2006). High-resolution radiation hybrid map of wheat chromosome 1D.. Genetics.

[11] Riera-Lizarazu O, Vales MI, Kianian SF (2008). Radiation hybrid (RH) and HAPPY mapping in plants.. Cytogenet Genome Res.

[12] Cox DR, Burmeister M, Price ER, Kim S, Myers RM (1990). Radiation hybrid mapping: a somatic cell genetic method for constructing high-resolution maps of mammalian chromosomes.. Science.

[13] Goss SJ, Harris H (1975). New method for mapping genes in human chromosomes.. Nature.

[14] Gyapay G, Schmitt K, Fizames C, Jones H, Vega-Czarny N (1996). A radiation hybrid map of the human genome.. Hum Mol Genet.

[15] Schuler GD, Boguski MS, Stewart EA, Stein LD, Gyapay G (1996). A gene map of the human genome.. Science.

[16] Walter MA, Spillett DJ, Thomas P, Weissenbach J, Goodfellow PN (1994). A method for constructing radiation hybrid maps of whole genomes.. Nat Genet.

[17] Guyon R, Senger F, Rakotomanga M, Sadequi N, Volckaert FA (2010). A radiation hybrid map of the European sea bass (Dicentrarchus labrax) based on 1581 markers: Synteny analysis with model fish genomes.. Genomics.

[18] Park CC, Ahn S, Bloom JS, Lin A, Wang RT (2008). Fine mapping of regulatory loci for mammalian gene expression using radiation hybrids.. Nat Genet.

[19] McCarthy LC, Terrett J, Davis ME, Knights CJ, Smith AL (1997). A first-generation whole genome-radiation hybrid map spanning the mouse genome.. Genome Res.

[20] Watanabe TK, Bihoreau MT, McCarthy LC, Kiguwa SL, Hishigaki H (1999). A radiation hybrid map of the rat genome containing 5,255 markers.. Nat Genet.

[21] Riera-Lizarazu O, Vales MI, Ananiev EV, Rines HW, Phillips RL (2000). Production and characterization of maize chromosome 9 radiation hybrids derived from an oat-maize addition line.. Genetics.

[22] Wardrop J, Fuller J, Powell W, Machray GC (2004). Exploiting plant somatic radiation hybrids for physical mapping of expressed sequence tags.. Theor Appl Genet.

[23] Wardrop J, Snape J, Powell W, Machray GC (2002). Constructing plant radiation hybrid panels.. Plant Journal.

[24] Gao W, Chen ZJ, Yu JZ, Kohel RJ, Womack JE (2006). Wide-cross whole-genome radiation hybrid mapping of the cotton (Gossypium barbadense L.) genome.. Mol Gen Genet.

[25] Gao W, Chen ZJ, Yu JZ, Raska D, Kohel RJ (2004). Wide-cross whole-genome radiation hybrid mapping of cotton (Gossypium hirsutum L.).. Genetics.

[26] Hossain KG, Kalavacharla V, Lazo GR, Hegstad J, Wentz MJ (2004). A chromosome bin map of 2148 expressed sequence tag loci of wheat homoeologous group 7.. Genetics.

[27] Xia G, Xiang F, Zhou A, Wang H, Chen H (2003). Asymmetric somatic hybridization between wheat (Triticum aestivum L.) and Agropyron elongatum (Host) Nevishi.. Theor Appl Genet.

[28] Xia G (2009). Progress of chromosome engineering mediated by asymmetric somatic hybridization.. J Genet Genomics.

[29] Bate GW, Hasenkampf CA, Contolini CL, Piastuch WC (1987). Asymmetric hybridization in *Nicotiana* by fusion of irradiated protoplasts.. Theor Appl Genet.

[30] Forsberg J, Dixelius C, Lagercrantz U, Glimelius K (1998). UV dose-dependent DNA elimilation in asymmetric somatic hybrids between Brassica napus and Arabidopsis thaliana.. Plant Science.

[31] Forsberg J, Lagercrantz U, Glimelius K (1998). Comparison of UV light, X-ray and restriction enzyme treatment as tools in production of asymmetric somatic hybrids between Brassica napus and Arabidopsis thaliana.. Theor Appl Genet.

[32] Ramulu KS, Dijkhuis P, Verhoeven HA, Famelaer I, Blaas J (1992). Microprotoplast isolation, enrichment and fusion for partial genome transfer in plants.. Physiologia Plantarum.

[33] Hinnisdaels S, Bariller L, Mouras A, Sidorov V, Del-Favero J (1991). Highly asymmetric intergeneric nuclear hybrids between Nicotiana and Petunia: evidence for recombination and translocation events in somatic hybrid plants after ‘gamma-fusion’.. Theor Appl Genet.

[34] Zubko K, Zubko I, Gleba Y (2002). Self-fertile cybrids Nicotiana tabacum (+ Hyoscyamus aureus) with a nucleo-plastome incompatibility.. Theor Appl Genet.

[35] Vlahova M, Hinnisdaels S, Frulleux F, Claeys M, Atanassov A (1997). UV irradiation as a tool for obtaining asymmetric somatic hybrids between Nicotiana plumbaginifolia and Lycoperdicon esculentum.. Theor Appl Genet.

[36] Xia GM, Li ZY, Guo GQ, Chen HM (1992). Direct somatic embryogenesis and plant regeneration from protoplasts of *Bupleurum scorzonerifolium* Wild.. Plant Cell Reports.

[37] Wang MQ, Xia GM, Peng ZY (2005). High UV-tolerance with introgression hybrid formation of *Bupleurum scorzonerifolium Willd* Plant Science.

[38] Zhou C, Xia G, Zhi D, Chen Y (2006). Genetic characterization of asymmetric somatic hybrids between Bupleurum scorzonerifolium Willd and Triticum aestivum L.: potential application to the study of the wheat genome.. Planta.

[39] Wang MQ, Zhao JS, Peng ZY, Guo W, Wang Y (2008). Chromosomes are eliminated in the symmetric fusion between *Arabidopsis thaliana* L. and *Bupleurum scorzonerifolium*.. Plant Cell, Tissue and Organ Culture.

[40] Xia GM, Chen HM (1996). Plant regeneration from intergeneric somatic hybridization between Trticum aestivum L and Leymus chinensis (Trin) Tzvel.. Plant Science.

[41] Zhou AF, Xia GM, Chen HM, Hu H (2001). Analysis of chromosomal and organellar DNA of somatic hybrids between Triticum aestiuvm L.and Haynaldia villosa Schru.. Mol Gen Genet.

[42] Panaud O, Chen X, McCouch SR (1996). Development of microsatellite markers and characterization of simple sequence length polymorphism (SSLP) in rice (*Oryza sativa* L.).. Mol Gen Genet.

[43] Breseghello F, Sorrells ME (2006). Association mapping of kernel size and milling quality in wheat (Triticum aestivum L.) cultivars.. Genetics.

[44] Gegas VC, Nazari A, Griffiths S, Simmonds J, Fish L (2010). A genetic framework for grain size and shape variation in wheat.. Plant Cell.

[45] Gill BS, Friebe B, Endo TR (1991). Standard karyotype and nomenclature system for description of chromosome bands and structural aberrations in wheat (*Triticum aestivum*).. Genome.

[46] Arumuganathan K, Earle ED (1991). Nuclear DNA content of some important plant species.. Plant Molecular Biology Reporter.

[47] Sourdille P, Singh S, Cadalen T, Brown-Guedira GL, Gay G (2004). Microsatellite-based deletion bin system for the establishment of genetic-physical map relationships in wheat (Triticum aestivum L.).. Functional & integrative genomics.

[48] Vitulo N, Albiero A, Forcato C, Campagna D, Dal Pero F (2011). First survey of the wheat chromosome 5A composition through a next generation sequencing approach.. PloS one.

[49] De Filippis L, Hoffmann E, Hampp R (1996). Identification of somatic hybrids of tobacco generated by electrofusion and culture of protoplasts using RAPD-PCR Plant Science.

[50] Hossain KG, Riera-Lizarazu O, Kalavacharla V, Vales MI, Maan SS (2004). Radiation hybrid mapping of the species cytoplasm-specific (scsae) gene in wheat.. Genetics.

[51] Ananiev EV, Riera-Lizarazu O, Rines HW, Phillips RL (1997). Oat-maize chromosome addition lines: a new system for mapping the maize genome.. Proc Natl Acad Sci U S A.

[52] Stewart EA, Cox DR, Dear PH (1997). Radiation hybrid mapping..

[53] Jones HB (1996). Hybrid selection as a method of increasing mapping power for radiation hybrids.. Genome Res.

[54] Linkiewicz AM, Qi LL, Gill BS, Ratnasiri A, Echalier B (2004). A 2500-locus bin map of wheat homoeologous group 5 provides insights on gene distribution and colinearity with rice.. Genetics.

