# Supplementary figures and images for: Construction of Whole Genome Radiation Hybrid Panels and Map of Chromosome 5A of Wheat Using Asymmetric Somatic Hybridization
Source: PLoS One. 2012 Jul 16;7(7):e40214. doi: 10.1371/journal.pone.0040214 (PMC3398029; doi:10.1371/journal.pone.0040214)

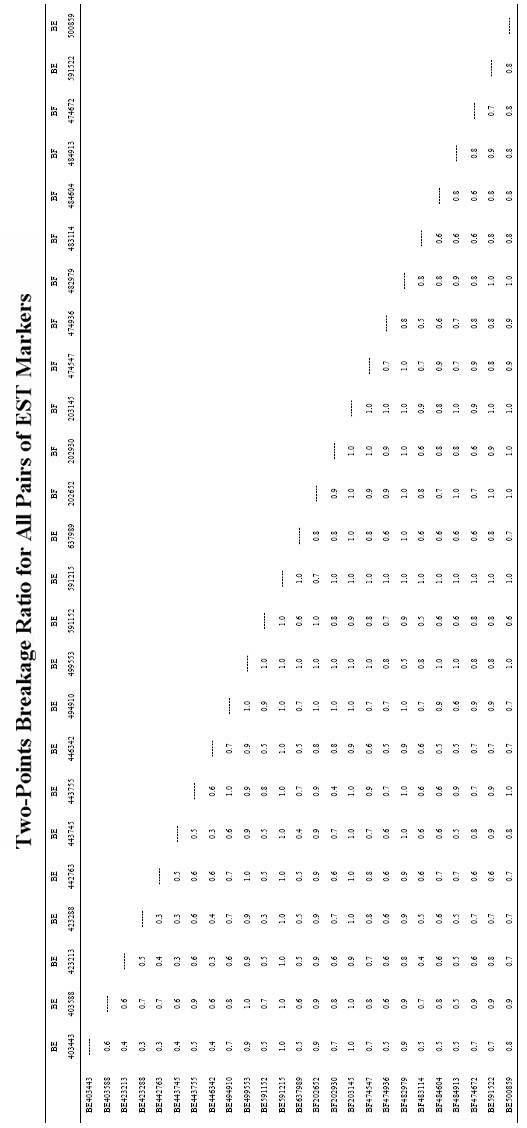


FIGURE S1. Two-points breakage ratio matrix of the 25 EST loci of bin C-5AL10-0.57*

Supplement: Figure S1 — Two-points breakage ratio matrix of the 25 EST loci of bin C-5AL10-0.57*. (DOCX) [file pone.0040214.s001.docx]
