# Supplementary material for: Construction of Whole Genome Radiation Hybrid Panels and Map of Chromosome 5A of Wheat Using Asymmetric Somatic Hybridization
Source: PLoS One. 2012 Jul 16;7(7):e40214. doi: 10.1371/journal.pone.0040214 (PMC3398029; doi:10.1371/journal.pone.0040214)
Supplement: Table S1 — The RH map of 68 microsatellite loci of wheat chromosome 5A. (DOCX) [file pone.0040214.s002.docx]

TABLE S1 The RH map of 68 microsatellite loci of wheat chromosome 5A

| Markers | | | Distance  (cR) | Cumulative  (cR) | Theta  (% age) | 2pt LOD |
| --- | --- | --- | --- | --- | --- | --- |
| No. |  | Name |  |  |  |  |
| 1 |  | Xwmc415 | 56.5 | 56.5 | 43.2 | 12.4 |
| 2 |  | Xbarc122 | 52.0 | 108.5 | 40.6 | 12.7 |
| 3 |  | Xgwm291 | 38.2 | 146.7 | 31.7 | 14.8 |
| 4 |  | Xgwm410 | 18.1 | 164.8 | 16.6 | 23.2 |
| 5 |  | Xgwm154 | 24.5 | 189.3 | 21.7 | 21.1 |
| 6 |  | Xwmc159 | 43.8 | 233.1 | 35.5 | 15.3 |
| 7 |  | Xbarc100 | 63.6 | 296.6 | 47.0 | 8.2 |
| 8 |  | Xgwm595 | 45.4 | 342.0 | 36.5 | 11.2 |
| 9 |  | Xbarc151 | 26.7 | 368.6 | 23.4 | 21.3 |
| 10 |  | Xbarc1 | 25.3 | 393.9 | 22.4 | 23.4 |
| 11 |  | Xgwm179 | 24.3 | 418.2 | 21.6 | 25.7 |
| 12 |  | Xwmc388 | 18.1 | 436.3 | 16.5 | 29.6 |
| 13 |  | Xwmc150 | 17.1 | 453.4 | 15.7 | 31.1 |
| 14 |  | Xcfa2104 | 27.2 | 480.6 | 23.8 | 25.9 |
| 15 |  | Xbarc1135 | 26.8 | 507.3 | 23.5 | 26.3 |
| 16 |  | Xgwm443 | 24.6 | 531.9 | 21.8 | 27.5 |
| 17 |  | Xcfa2121 | 26.4 | 558.3 | 23.2 | 25.8 |
| 18 |  | Xwmc445 | 38.1 | 596.4 | 31.7 | 19.9 |
| 19 |  | Xbarc144 | 39.1 | 635.5 | 32.4 | 19.6 |
| 20 |  | Xwmc327 | 35.2 | 670.7 | 29.6 | 21.2 |
| 21 |  | Xwmc47 | 38.1 | 708.8 | 31.7 | 20.2 |
| 22 |  | Xcfa2250 | 33.1 | 741.9 | 28.2 | 22.0 |
| 23 |  | Xgwm666 | 33.5 | 775.5 | 28.5 | 21.5 |
| 24 |  | Xwmc492 | 35.8 | 811.2 | 30.1 | 21.1 |
| 25 |  | Xbarc135 | 25.5 | 836.7 | 22.5 | 26.2 |
| 26 |  | Xgwm126 | 18.4 | 855.2 | 16.8 | 29.9 |
| 27 |  | Xcfa2163 | 27.8 | 883.0 | 24.3 | 23.6 |
| 28 |  | Xgwm205 | 25.6 | 908.6 | 22.6 | 25.8 |
| 29 |  | Xgdm109 | 15.6 | 924.1 | 14.4 | 32.9 |
| 30 |  | Xbarc94 | 24.2 | 948.3 | 21.5 | 27.0 |
| 31 |  | Xwmc727 | 29.3 | 977.6 | 25.4 | 24.9 |
| 32 |  | Xbarc230 | 29.8 | 1007.4 | 25.8 | 24.6 |
| 33 |  | Xbarc1182 | 34.5 | 1041.9 | 29.2 | 21.9 |
| 34 |  | Xcfa2190 | 25.4 | 1067.3 | 22.4 | 24.7 |
| 35 |  | Xbarc10 | 31.4 | 1098.7 | 27.0 | 20.8 |
| 36 |  | Xwmc577 | 21.9 | 1120.6 | 19.6 | 26.9 |
| 37 |  | Xbarc316 | 24.7 | 1145.2 | 21.9 | 25.5 |
| 38 |  | Xbarc319 | 27.7 | 1172.9 | 24.2 | 23.7 |
| 39 |  | Xgwm186 | 9.5 | 1182.4 | 9.1 | 35.5 |
| 40 |  | Xwmc475 | 9.4 | 1191.8 | 9.0 | 33.5 |
| 41 |  | Xcfa2155 | 32.5 | 1224.3 | 27.7 | 18.5 |
| 42 |  | Xbarc155 | 31.9 | 1256.2 | 27.3 | 19.6 |
| 43 |  | Xbarc180 | 22.2 | 1278.4 | 19.9 | 24.6 |
| 44 |  | Xbarc56 | 14.3 | 1292.7 | 13.3 | 29.5 |
| 45 |  | Xwmc524 | 21.1 | 1313.8 | 19.0 | 25.6 |
| 46 |  | Xbarc92 | 17.5 | 1331.3 | 16.0 | 29.7 |
| 47 |  | Xgwm129 | 24.7 | 1356.1 | 21.9 | 25.6 |
| 48 |  | Xwmc110 | 26.3 | 1382.4 | 23.1 | 24.3 |
| 49 |  | Xgwm415 | 24.8 | 1407.2 | 22.0 | 24.7 |
| 50 |  | Xgwm156 | 29.8 | 1437.0 | 25.8 | 21.9 |
| 51 |  | Xbarc186 | 56.5 | 1493.5 | 43.1 | 12.7 |
| 52 |  | Xbarc197 | 93.8 | 1587.3 | 60.9 | 5.9 |
| 53 |  | Xwmc713 | 52.5 | 1639.8 | 40.9 | 14.0 |
| 54 |  | Xcfa2141 | 44.4 | 1684.3 | 35.9 | 17.2 |
| 55 |  | Xbarc1158 | 26.5 | 1710.8 | 23.3 | 25.9 |
| 56 |  | Xgwm617 | 25.4 | 1736.1 | 22.4 | 25.8 |
| 57 |  | Xbarc141 | 40.2 | 1776.4 | 33.1 | 19.1 |
| 58 |  | Xbarc115 | 27.3 | 1803.7 | 23.9 | 25.3 |
| 59 |  | Xgwm234 | 23.4 | 1827.1 | 20.9 | 27.0 |
| 60 |  | Xwmc705 | 18.7 | 1845.8 | 17.1 | 29.8 |
| 61 |  | Xbarc165 | 21.1 | 1866.9 | 19.0 | 27.3 |
| 62 |  | Xgwm639 | 26.3 | 1893.3 | 23.1 | 23.7 |
| 63 |  | Xgwm293 | 35.0 | 1928.2 | 29.5 | 19.6 |
| 64 |  | Xbarc117 | 29.0 | 1957.2 | 25.2 | 23.3 |
| 65 |  | Xwmc452 | 39.4 | 1996.6 | 32.6 | 19.1 |
| 66 |  | Xgwm6 | 46.0 | 2042.6 | 36.9 | 17.2 |
| 67 |  | Xgwm304 | 60.4 | 2103.0 | 45.3 | 12.5 |
| 68 |  | Xwmc446 | ――― | ――― | ――― | ――― |
