# Supplementary material for: Construction of Whole Genome Radiation Hybrid Panels and Map of Chromosome 5A of Wheat Using Asymmetric Somatic Hybridization
Source: PLoS One. 2012 Jul 16;7(7):e40214. doi: 10.1371/journal.pone.0040214 (PMC3398029; doi:10.1371/journal.pone.0040214)
Supplement: Table S2 — The putative rice homologs of 25 ESTs of bin C-5AL10-0.57*. (DOCX) [file pone.0040214.s003.docx]

TABLE S2 The putative rice homologues of 25 ESTs within the bin C-5AL10-0.57*

|  | **EST** | **Hit score** | **E-  value** | **Top query coverage** | **Rice homolog** | **Rice chr.** | **Location** | **Description** |
| --- | --- | --- | --- | --- | --- | --- | --- | --- |
| 1 | BF202652 | 802 | 8.2e-80 | 96.64% | [LOC_Os09g37100.1](http://rice.plantbiology.msu.edu/cgi-bin/ORF_infopage.cgi?orf=LOC_Os09g37100.1) | Chr9 | 21391774 - 21397949 | Phospholipase |
| 2 | BE403443 | 114 | 6.7e-05 | 33.51% | [LOC_Os10g19270.1](http://rice.plantbiology.msu.edu/cgi-bin/ORF_infopage.cgi?orf=LOC_Os10g19270.1) | Chr10 | 9816684 - 9813592 | retrotransposon protein |
| 3 | BE403588 | 62 | 0.42 | 4.53% | [LOC_Os10g19898.1](http://rice.plantbiology.msu.edu/cgi-bin/ORF_infopage.cgi?orf=LOC_Os10g19898.1) | Chr10 | 9959380 - 9957415 | hypothetical protein |
| 4 | BE423213 | 274 | 7.3e-24 | 44.38% | [LOC_Os09g37006.2](http://rice.plantbiology.msu.edu/cgi-bin/ORF_infopage.cgi?orf=LOC_Os09g37006.2) | Chr9 | 21346652 - 21343532 | Chloroplast nucleoid DNA-binding protein-like protein |
| 5 | BE423288 | 241 | 2.3e-20 | 55.79% | [LOC_Os06g07941.1](http://rice.plantbiology.msu.edu/cgi-bin/ORF_infopage.cgi?orf=LOC_Os06g07941.1) | Chr6 | 3858434 - 3860468 | iron/ascorbate-dependent oxidoreductase |
| 6 | BE442763 | 770 | 2.0e-76 | 78.07% | [LOC_Os02g32110.1](http://rice.plantbiology.msu.edu/cgi-bin/ORF_infopage.cgi?orf=LOC_Os02g32110.1) | Chr2 | 18976369 - 18972815 | exostosin family domain containing protein, putative |
| 7 | BE443745 | 710 | 3.5e-119 | 61.11% | [LOC_Os09g31270.1](http://rice.plantbiology.msu.edu/cgi-bin/ORF_infopage.cgi?orf=LOC_Os09g31270.1) | Chr9 | 18803450 - 18801144 | polygalacturonase, putative |
| 8 | BE443755 | 77 | 0.89 | 19.83% | [LOC_Os07g31244.1](http://rice.plantbiology.msu.edu/cgi-bin/ORF_infopage.cgi?orf=LOC_Os07g31244.1) | Chr7 | 18497822 - 18496787 | expressed protein |
| 9 | BE446342 | 711 | 5.8e-43 | 34.18% | [LOC_Os07g30170.1](http://rice.plantbiology.msu.edu/cgi-bin/ORF_infopage.cgi?orf=LOC_Os07g30170.1) | Chr7 | 17823755 - 17820555 | nitrilase, putative |
| 10 | BE494910 | 701 | 4.0e-69 | 96.90% | [LOC_Os03g04470.1](http://rice.plantbiology.msu.edu/cgi-bin/ORF_infopage.cgi?orf=LOC_Os03g04470.1) | Chr3 | 2073418 - 2075029 | expressed protein |
| 11 | BE499553 | NONE | NONE | NONE | NONE | NONE | NONE | NONE |
| 12 | BE591152 | 187 | 7.1e-14 | 41.36% | [LOC_Os05g41390.1](http://rice.plantbiology.msu.edu/cgi-bin/ORF_infopage.cgi?orf=LOC_Os05g41390.1) | Chr5 | 24240401 - 24243654 | cyclin, putative |
| 13 | BE591215 | 846 | 1.8e-84 | 9.84% | [LOC_Os07g14590.1](http://rice.plantbiology.msu.edu/cgi-bin/ORF_infopage.cgi?orf=LOC_Os07g14590.1) | Chr7 | 8325068 - 8321642 | hydrolase, putative, |
| 14 | BE637989 | 627 | 2.9e-61 | 96.08% | [LOC_Os12g01430.1](http://rice.plantbiology.msu.edu/cgi-bin/ORF_infopage.cgi?orf=LOC_Os12g01430.1) | Chr12 | 270166 - 267657 | ribosomal protein L10, putative |
| 15 | BE500859 | 95 | 6.8e-05 | 21.36% | [LOC_Os03g49450.1](http://rice.plantbiology.msu.edu/cgi-bin/ORF_infopage.cgi?orf=LOC_Os03g49450.1) | Chr3 | 28149008 - 28149238 | expressed protein |
| 16 | BE591522 | 646 | 2.8e-63 | 96.09% | [LOC_Os09g29890.1](http://rice.plantbiology.msu.edu/cgi-bin/ORF_infopage.cgi?orf=LOC_Os09g29890.1) | Chr9 | 18178939 - 18174532 | phosphatidylinositol 3- and 4-kinase family protein, putative |
| 17 | BF474672 | 69 | 0.094 | z | [LOC_Os05g23393.1](http://rice.plantbiology.msu.edu/cgi-bin/ORF_infopage.cgi?orf=LOC_Os05g23393.1) | Chr5 | 13366937 - 13367660 | expressed protein |
| 18 | BF484913 | 867 | 1.1e-86 | 99.46% | [LOC_Os09g20820.1](http://rice.plantbiology.msu.edu/cgi-bin/ORF_infopage.cgi?orf=LOC_Os09g20820.1) | Chr9 | 12544907 - 12540619 | enolase, putative |
| 19 | BF484604 | 277 | 1.3e-23 | 72.45% | [LOC_Os09g20560.1](http://rice.plantbiology.msu.edu/cgi-bin/ORF_infopage.cgi?orf=LOC_Os09g20560.1) | Chr9 | 12389176 - 12392355 | pumilio-family RNA binding repeat containing protein |
| 20 | BF483114 | 72 | 0.999 | 10.66% | [LOC_Os08g40790.2](http://rice.plantbiology.msu.edu/cgi-bin/ORF_infopage.cgi?orf=LOC_Os08g40790.2) | Chr8 | 25804334 - 25799137 | DNA repair ATPase-related, putative |
| 21 | BF482979 | 423 | 1.2e-39 | 87.02% | [LOC_Os03g58470.1](http://rice.plantbiology.msu.edu/cgi-bin/ORF_infopage.cgi?orf=LOC_Os03g58470.1) | Chr3 | 33304497 - 33305990 | retrotransposon protein, putative |
| 22 | BF203145 | 764 | 8.7e-76 | 99.56% | [LOC_Os05g35480.1](http://rice.plantbiology.msu.edu/cgi-bin/ORF_infopage.cgi?orf=LOC_Os05g35480.1) | Chr5 | 21084058 - 21080988 | anthranilate phosphoribosyltransferase, putative |
| 23 | BF202930 | 5 | 0.0019 | 36.28% | [LOC_Os04g32404.1](http://rice.plantbiology.msu.edu/cgi-bin/ORF_infopage.cgi?orf=LOC_Os04g32404.1) | Chr4 | 19435390 - 19430045 | expressed protein |
| 24 | BF474936 | 491 | 3.5e-51 | 5.29% | [LOC_Os12g18120.2](http://rice.plantbiology.msu.edu/cgi-bin/ORF_infopage.cgi?orf=LOC_Os12g18120.2) | Chr12 | 10444026 - 10436088 | zinc finger C-x8-C-x5-C-x3-H type family protein |
| 25 | BF474547 | 110 | 2.6e-08 | 67.67% | [LOC_Os04g31610.1](http://rice.plantbiology.msu.edu/cgi-bin/ORF_infopage.cgi?orf=LOC_Os04g31610.1) | Chr4 | 18907157 - 18905455 | OsFBO3 - F-box and other domain containing protein |
