# Supplementary material for: Construction of Whole Genome Radiation Hybrid Panels and Map of Chromosome 5A of Wheat Using Asymmetric Somatic Hybridization
Source: PLoS One. 2012 Jul 16;7(7):e40214. doi: 10.1371/journal.pone.0040214 (PMC3398029; doi:10.1371/journal.pone.0040214)
Supplement: Table S3 — The RH map of 46 ESTs of wheat chromosome 5AL. (DOCX) [file pone.0040214.s004.docx]

TABLE S3 The RH map of 46 ESTs of wheat chromosome 5AL

| Markers | | | Distance  (cR) | Cumulative  (cR) | Theta  (% age) | 2pt LOD |
| --- | --- | --- | --- | --- | --- | --- |
| No. |  | Name |  |  |  |  |
| 1 |  | BQ169441 | 19.3 | 19.3 | 15.4 | 26.3 |
| 2 |  | BQ170616 | 28.1 | 47.4 | 25.4 | 19.1 |
| 3 |  | BE497368 | 45.4 | 92.7 | 39.5 | 26.4 |
| 4 |  | BF145701 | 44.2 | 136.9 | 25.8 | 18.1 |
| 5 |  | BE494102 | 18.4 | 155.3 | 12.4 | 28.8 |
| 6 |  | BE403679 | 19.4 | 174.7 | 26.9 | 22.2 |
| 7 |  | BE404341 | 41.5 | 216.2 | 37.6 | 25.4 |
| 8 |  | BG605186 | 35.6 | 251.8 | 28.6 | 30.9 |
| 9 |  | BQ169704 | 39.1 | 290.9 | 27.4 | 29.1 |
| 10 |  | BM138656 | 24.3 | 315.2 | 15.5 | 19.8 |
| 11 |  | BF483937 | 32.5 | 347.8 | 26.6 | 21.0 |
| 12 |  | BE442918 | 46.3 | 394.1 | 35.9 | 21.4 |
| 13 |  | BE495790 | 23.2 | 417.2 | 28.5 | 31.6 |
| 14 |  | BE591522 | 27.2 | 444.4 | 34 | 22.3 |
| 15 |  | CD452735 | 15.7 | 460.1 | 44.5 | 35.2 |
| 16 |  | BE591912 | 31.2 | 491.3 | 37.3 | 23.5 |
| 17 |  | BF291958 | 12.2 | 503.5 | 19.9 | 21.7 |
| 18 |  | BE406463 | 35.5 | 539.0 | 35.8 | 26.3 |
| 19 |  | BE443925 | 27.5 | 566.5 | 23.6 | 19.1 |
| 20 |  | BQ166830 | 32.4 | 598.9 | 24.6 | 26.4 |
| 21 |  | BE498757 | 29.7 | 628.6 | 33.9 | 18.1 |
| 22 |  | BF201857 | 20.7 | 649.3 | 25.3 | 28.8 |
| 23 |  | BF484818 | 23.2 | 672.5 | 20.1 | 22.2 |
| 24 |  | BF482595 | 25.7 | 698.2 | 19.3 | 25.4 |
| 25 |  | BG313955 | 19.3 | 717.5 | 30.1 | 30.9 |
| 26 |  | BQ162095 | 31.4 | 748.9 | 20.6 | 29.1 |
| 27 |  | BE352573 | 20.8 | 769.7 | 24.3 | 19.8 |
| 28 |  | BE637338 | 29.3 | 799.0 | 25.5 | 21.0 |
| 29 |  | BF485285 | 21.2 | 820.2 | 19.4 | 21.4 |
| 30 |  | CD454046 | 25.7 | 845.9 | 25.7 | 31.6 |
| 31 |  | BF474090 | 16.6 | 862.5 | 11.2 | 22.3 |
| 32 |  | CD490668 | 28.3 | 890.8 | 17.2 | 22.9 |
| 33 |  | BG606780 | 20.1 | 910.9 | 27.2 | 29.4 |
| 34 |  | CD454129 | 26.9 | 937.8 | 23.2 | 27.6 |
| 35 |  | BE404341 | 21.2 | 959.0 | 22.9 | 17.3 |
| 36 |  | BQ172317 | 14.4 | 973.4 | 12.7 | 21.0 |
| 37 |  | BE497820 | 25.7 | 999.2 | 16 | 18.4 |
| 38 |  | BE604883 | 11.2 | 1010.4 | 37.7 | 21.6 |
| 39 |  | BE494426 | 15.6 | 1026.0 | 26.6 | 19.1 |
| 40 |  | BE425968 | 24.1 | 1050.1 | 28.3 | 18.7 |
| 41 |  | CD492057 | 25.4 | 1075.4 | 21.3 | 22.6 |
| 42 |  | BQ172265 | 30.7 | 1106.1 | 15.4 | 28.0 |
| 43 |  | BQ171805 | 21.4 | 1127.5 | 13.3 | 26.0 |
| 44 |  | BE495011 | 54.3 | 1181.8 | 24.2 | 25.3 |
| 45 |  | BG313693 | 31.8 | 1213.6 | 22.4 | 22.4 |
| 46 |  | BE405706 | ――― | ――― | ――― | ――― |
|  |  |  |  |  |  |  |

The ESTs marked by red color show the inconsistent gene order with the virtual gene order.
